# Lymphopenia and lymphocytosis in practical healthy people born and living in the North

**DOI:** 10.1002/iid3.308

**Published:** 2020-05-16

**Authors:** Veronika P. Patrakeeva, Liliya K. Dobrodeeva

**Affiliations:** ^1^ N. Laverov Federal Center for Integrated Arctic Research, Russian Academy of Sciences, Institute of Environmental Physiology, Department of Environmental Immunology Arkhangelsk Russian Federation

**Keywords:** G‐CSF, lymphocytosis, lymphopenia, neutropenia, NFATc1

## Abstract

**Introduction:**

The purpose of this study was to elucidate the mechanisms of the formation of lymphopenia and lymphocytosis in healthy people, who are living and working in the Arctic region.

**Materials and Methods:**

A total of 88 practically healthy people living and working in the Arctic region were examined. An analysis of the results was carried out, depending on the concentration of lymphocytes in the peripheral venous blood: group 1—with lymphopenia, the content of lymphocytes below 1.5 × 10^9^cl/L (21 people); group 2—with a normal lymphocyte content from 1.5 to 3.5 × 10^9^cl/L (47 people); and group 3—with lymphocytosis, lymphocytes in the peripheral blood of more than 3.5 × 10^9^cl/L (20 people).

**Results:**

It has been established that the main mechanism for the formation of lymphopenia in a person living in the Arctic is the activation of the migration of functionally active lymphocytes in the tissue. The decrease in the number of circulating lymphocytes is a consequence of their redistribution from the circulating pool to the marginal one and an increase in the activity of adhesive molecules, in particular, the selectin ligand. It was revealed that an increase in the content of lymphocytes in the blood occurs upon the activation of the intracellular energy‐intensive mechanisms of lymphoproliferation with an increase in the consumption of intracellular ATP and the participation of the nuclear factor of activated T cells 1. It was shown that the restoration of the circulating pool of mature neutrophils is ensured by the principle of reverse regulation in response to neutropenia by stimulating granulocyte‐colony stimulating factor granulopoiesis.

**Conclusions:**

The main mechanism for the formation of lymphopenia and lymphocytosis in healthy people was established.

## Graphical abstract

In the study of the mechanisms of the formation of lymphopenia and lymphocytosis in healthy people, it has been established that the main mechanism for the formation of lymphopenia in a person living in the Arctic is the activation of the migration of functionally active lymphocytes in the tissue.

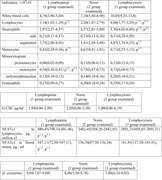

## 1 INTRODUCTION

A change in the content of lymphocytes (lymphopenia or lymphocytosis) in the peripheral blood can be recorded both in the presence of pathological changes in the body and in practically healthy people, without any diagnosed proliferative disorder or acute disease. Therefore, asymptomatic lymphopenia is often (17%‐32%) detected in people living in regions with extreme climates and adverse environmental conditions.^1^ The frequency of registration of asymptomatic lymphopenia increases during the polar night. However, the mechanisms of lymphopenia and lymphocytosis formation in practically healthy people have not been studied. A lot of work has been performed on the study of lymphopenia in pathological conditions. It is known that causes of lymphopenia can be different, including hereditary severe combined immunodeficiency,^2^ viral and bacterial infections,^3^, ^4^, ^5^, ^6^, ^7^, ^8^, ^9^, ^10^, ^11^ autoimmune diseases,^12^, ^13^, ^14^ malignant neoplasms,^15^, ^16^ taking medication,^17^ etc. The mechanisms for reducing the concentration of circulating lymphocytes can also be different, including the activation of apoptosis and cell necrosis, increased migration to tissues, decreased DNA synthesis and proliferation, the inhibition of their exit from lymphoid tissues, increased sensitivity of lymphocytes to complement‐mediated cytolysis,^18^ impaired maturation, and the differentiation of lymphocytes.^19^

The purpose of this study was to elucidate the mechanisms of the formation of lymphopenia and lymphocytosis in healthy people.

## 2 MATERIALS AND METHODS

A survey of 88 practically healthy people living and working in the village of Revda in the Murmansk region was conducted. The subjects were practically healthy and had no chronic and/or recurrent diseases. Blood was taken on an empty stomach in the morning from 8:00 to 10:00 am. All studies were performed with informed consent and carried out in compliance with the ethical standards set forth in the Helsinki Declaration and European Community Directives (8/609ES). An analysis of the results was carried out, depending on the concentration of lymphocytes in the peripheral venous blood: group 1—with lymphopenia, the content of lymphocytes below 1.5 × 10^9^cl/L (21 people); group 2—with a normal lymphocyte content from 1.5 to 3.5 × 10^9^cl/L (47 people); and group 3—with lymphocytosis, lymphocytes in the peripheral blood of more than 3.5 × 10^9^cl/L (20 people). In the peripheral blood, a leukogram was determined on an XS‐1000i hematology analyzer (Sysmex, Japan). In blood smears stained according to Romanovsky‐Giemsa, a lymphocytogram was counted (according to the method of IA Kassirsky, 1970),^20^ and a monocytogram (according to the method of ON Grigorova, 1956).^21^ By ELISA, serum nuclear factor of activated T cells 1 (NFATc1) (Cusabio), granulocyte‐colony stimulating factor (G‐CSF) (eBioscience), and sL‐selectin (eBioscience) were determined on Multiscan MS. Using ELISA, lymphocyte lysate was used to determine NFATc1 (Cusabio) on Multiscan MS. The research results were processed using the software package Statistica 6 (StatSoft, Tulsa, OK). The Mann‐Whitney test was used to determine the statistical hypothesis of the difference in values. When analyzing the results, we used the median, lower, and upper quartiles (M [Q1‐Q3]). The statistical significance of the differences between the groups was evaluated using the nonparametric Wilcoxon test. The critical level of significance (*P*) in the work was found to be equal to .017.

## 3 RESULTS AND DISCUSSION

Among the examined patients, lymphopenia was recorded in 23.86%, where the peripheral venous blood lymphocyte count is less than 1.5 × 10^9^cl/L, 53.41% had normal lymphocyte counts and lymphocytosis was recorded in 22.73% of cases, that is, an increase in the level of lymphocytes above 3.5 × 10^9^cl/L. Thus, three comparison groups were distinguished (Table 1).

**Table 1:** Indicators of leukogram in people, depending on the content of lymphocytes in peripheral blood, M (25‐75)

	Lymphopenia	Norm	Lymphocytosis
Indicators, ×10^9^cl/L	(group 1 examined)	(group 2 examined)	(group 3 examined)
White blood cells	4.70 (3.90‐5.60)	7.10 (5.80‐8.90)	10.05 (9.25‐13.8)
Lymphocytes	1.14 (1.02‐1.29) р^1‐2^	2.20 (1.87‐2.79)	4.06 (3.77‐5.29) р^1‐3^, р^2‐3^
Neutrophils	2.97 (2.27‐4.37)	3.57 (2.81‐5.80)	5.30 (4.02‐6.60), р^1‐3^, р^2‐3^
Stab	0.21 (0.11‐0.37)	0.23 (0.12‐0.36)	0.31 (0.20‐0.50)
Segmented	2.75 (2.06‐4.03)	3.41 (2.49‐5.48)	4.87 (3.74‐6.25), р^1‐3^
Monocytes	0.42 (0.28‐0.56), р^1‐2^	0.67 (0.41‐1.01)	0.71 (0.23‐1.13), р^1‐3^
Monocytogram			
Promonocytes	0.08 (0.02‐0.09)	0.11 (0.06‐0.11)	0.13 (0.12‐0.15)
Monocytes	0.39 (0.36‐0.41) р^1‐2^, р^1‐3^	0.73 (0.47‐0.73)	0.73 (0.68‐0.73)
Polymorphonuclear	0.12 (0.10‐0.15)	0.14 (0.10‐0.16)	0.20 (0.18‐0.21)
Eosinophils	0.17 (0.09‐0.27)	0.20 (0.10‐0.24)	0.25 (0.17‐0.26)

As can be seen from the data presented, lymphopenia in healthy people is associated with a decrease in the content of monocytes. This decrease occurs due to functionally active mature cells, while the concentrations of promonocytes and polymorphonuclear cells do not change. It is likely that such a decrease in peripheral blood monocytes is associated with the need to increase the activity of tissue monocytes and increase their migration. In people with normal lymphocyte counts, as well as in individuals with lymphocytosis, the levels of monocytes in the peripheral blood do not actually differ and monocytosis is detected in 46.8% and 50.2%, respectively.

Lymphopenia is combined with a lower neutrophil content, which is ensured by a reduction in the circulation of mature segmented cells, while the levels of stab forms are not significantly different (Table 1). Against the background of an increase in the level of neutrophilic granulocytes, the concentration of G‐CSF in the peripheral blood is reduced by a factor of 2.8 (Table 2).

**Table 2:** The concentration of G‐CSF in serum, depending on the content of lymphocytes in peripheral blood, M (25‐75)

	Lymphopenia	Norm	Lymphocytosis
	(group 1 examined)	(group 2 examined)	(group 3 examined)
G‐CSF, pg/mL	2.99 (0.06‐2.99)	2.05 (0.06‐5.30)	1.08 (0.06‐6.19)

Abbreviation: G‐CSF, granulocyte‐colony stimulating factor.

The concentration of mature neutrophils at the periphery and the level of G‐CSF is regulated by the feedback principle; an increase in the content of neutrophils at the periphery leads to accelerated decay of G‐CSF and, accordingly, a decrease in its concentration. G‐CSF effectively stimulates colony formation from a more mature population of neutrophil precursor cells in the bone marrow. It is possible that the role of G‐CSF in the formation of lymphocytosis is not limited to the action of this growth factor on neutrophils. The function of G‐CSF in peripheral blood is not completely determined, although it is known that virtually any cell in the body is capable of synthesizing this growth factor; the largest number of G‐CSF receptors is found in mature neutrophils and their number increases with the level of cell maturation.^22^ The effect on various types of cells is also mixed. In neutrophils, G‐CSF does not cause a significant change in the intracellular concentration of Ca^2+^ or pH,^23^ while, when acting on endothelial cells, it is able to dramatically increase intracellular pH and activate Na^+^/H^+^ metabolism.^24^

The decrease in lymphocyte count is associated with the accumulation of NFATc1 in serum. The appearance of this nuclear activation factor outside of the cell is probably a consequence of the activation of lymphocyte apoptosis, which is confirmed by a higher content of cells with the CD95 marker for lymphopenia (0.53 ± 0.05 × 10^9^cl/L) compared with the state of lymphocytosis (0.45 ± 0.04 × 10^9^cl/L) (Table 3). In parallel with the increase in lymphocyte count, the accumulation of intracellular NFATc1 is recorded, which indicates the inclusion of intracellular activation mechanisms. Proteins of the NFAT family affect lymphocyte activation processes, regulation of the cell cycle and apoptosis, as well as cytokine expression.^25^ NFATc1 controls the expression of transcription factors interferon regulatory factor 4, hypoxia‐inducible factor 1 regulating glycolysis, and mitochondrial respiration in T cells. In this way, NFATc1 can control the differentiation of T cells through their metabolic reprogramming.^26^, ^27^, ^28^ This is consistent with our earlier data showing that the ATP level decreases with an increase in the content of lymphocytes in the peripheral blood (2.38 ± 0.44 μmol/million.cL with lymphopenia and 0.58 ± 0.81 μmol/million.cL with lymphocytosis; *Р *< .001).^29^ This is probably due to its increased consumption in the process of the activation and differentiation of cells with the participation of NFATc1. On the other hand, as a result of prolonged chronic stimulation, the accumulation of NFATc1 in the cell can lead to the formation of anergic CD4+ and CD8+ T cells.^30^, ^31^, ^32^

**Table 3:** Level of nuclear marker of NFATc1 activation in lymphocytes and in blood serum, M (25‐75)

	Lymphopenia	Norm	Lymphocytosis
	(group 1 examined)	(group 2 examined)	(group 3 examined)
NFATc1 in lymphocytes, pg/million cL	880.45 (798.10‐881.46), р^1‐2^, р^1‐3^	2482.45(504.20‐2482.45)	2891.21 (605.65‐2891.21)
NFATc1 in blood serum, pg/mL	547.11 (72.50‐547.11), р^1‐2^, р^1‐3^	136.34 (87.50‐136.34)	181.91 (117.50‐181.91)

Abbreviation: NFATc1, nuclear factor of activated T cells 1.

A change in the level of the content of adhesive molecules can affect the content of cells in the circulation. Therefore, at low concentrations of lymphocytes in the peripheral blood, higher concentrations of sL‐selectin are detected (Table 4). Elevated levels of sL‐selectin are recorded in various pathologies (ulcerative colitis, insulin‐dependent diabetes mellitus, HIV infection, etc). The effect of sL‐selectin is to activate and attract white blood cells to the site of inflammation, while acting as an intercellular signaling molecule, sL‐selectin activates other adhesion molecules—integrins, peptides of the immunoglobulin superfamily. Thus, registered lymphopenia may be the result of an increase in the activity of adhesive molecules, in particular sL‐selectin, which triggers the activation mechanism of leukocyte adhesion and their advancement in tissue.

**Table 4:** The content of sL‐selectin in peripheral blood, depending on the content of lymphocytes in peripheral blood, M (25‐75)

	Lymphopenia	Norm	Lymphocytosis
	(group 1 examined)	(group 2 examined)	(group 3 examined)
sL‐selectin	9.69 (7.87‐9.69)	8.48 (5.50‐9.78)	7.89 (6.10‐9.02)

## 4 CONCLUSIONS

1. A decrease in the content of lymphocytes is associated with a decrease in the number of mature monocytes and neutrophils in the peripheral blood. Given the sequence of the formation of a preventive inflammation, which is part of the immune response, lymphopenia is most likely formed against the background of a deficiency of neutrophilic granulocytes and monocytes in the circulation. The main mechanism for the formation of lymphopenia in a person living in the Arctic is to activate the migration of functionally active lymphocytes into the tissue. The decrease in the number of circulating lymphocytes is a consequence of their redistribution from the circulating pool to the marginal one and an increase in the activity of adhesive molecules, in particular, the selectin ligand, which increases the relationship between cells and the endothelium. Participation in this process of apoptosis is not excluded, as evidenced by an increase in the content of extracellular NFATc1.
2. An increase in the content of lymphocytes in the blood occurs upon activation of the intracellular energy‐intensive mechanisms of lymphoproliferation with an increase in the consumption of intracellular ATP and the participation of NFATc1.
3. Recovery of the circulating pool of mature neutrophils is ensured by the principle of reverse regulation in response to neutropenia by stimulating G‐CSF granulopoiesis.

## CONFLICT OF INTERESTS

The authors declare that there are no conflict of interests.

## Data Availability

The data generated within the study is shown in this manuscript. Any raw data or analysis would be available from the corresponding author upon request.
